# Whole genome sequencing and function prediction of 133 gut anaerobes isolated from chicken caecum in pure cultures

**DOI:** 10.1186/s12864-018-4959-4

**Published:** 2018-07-31

**Authors:** Matej Medvecky, Darina Cejkova, Ondrej Polansky, Daniela Karasova, Tereza Kubasova, Alois Cizek, Ivan Rychlik

**Affiliations:** 1 Veterinary Research Institute, Hudcova 70, 621 00 Brno, Czech Republic; 2 Central European Institute of Technology (CEITEC), University of Veterinary and Pharmaceutical Sciences Brno, Brno, Czech Republic; 3 Department of Infectious Diseases and Microbiology, Faculty of Veterinary Medicine, University of Veterinary and Pharmaceutical Sciences Brno, Brno, Czech Republic

**Keywords:** Microbiota, Microbiome, Chicken, Whole genome sequencing, Metabolic pathway, Host-microbiota interactions, Anaerobe, Butyrate, Propionate

## Abstract

**Background:**

In order to start to understand the function of individual members of gut microbiota, we cultured, sequenced and analysed bacterial anaerobes from chicken caecum.

**Results:**

Altogether 204 isolates from chicken caecum were obtained in pure cultures using Wilkins-Chalgren anaerobe agar and anaerobic growth conditions. Genomes of all the isolates were determined using the NextSeq platform and subjected to bioinformatic analysis. Among 204 sequenced isolates we identified 133 different strains belonging to seven different phyla - *Firmicutes*, *Bacteroidetes*, *Actinobacteria*, *Proteobacteria*, *Verrucomicrobia*, *Elusimicrobia* and *Synergistetes*. Genome sizes ranged from 1.51 Mb in *Elusimicrobium minutum* to 6.70 Mb in *Bacteroides ovatus*. Clustering based on the presence of protein coding genes showed that isolates from phyla *Proteobacteria*, *Verrucomicrobia*, *Elusimicrobia* and *Synergistetes* did not cluster with the remaining isolates. *Firmicutes* split into families *Lactobacillaceae*, *Enterococcaceae*, *Veillonellaceae* and order *Clostridiales* from which the *Clostridium perfringens* isolates formed a distinct sub-cluster. All *Bacteroidetes* isolates formed a separate cluster showing similar genetic composition in all isolates but distinct from the rest of the gut anaerobes. The majority of *Actinobacteria* clustered closely together except for the representatives of genus *Gordonibacter* showing that the genome of this genus differs from the rest of *Actinobacteria* sequenced in this study. Representatives of *Bacteroidetes* commonly encoded proteins (collagenase, hemagglutinin, hemolysin, hyaluronidase, heparinases, chondroitinase, mucin-desulfating sulfatase or glutamate decarboxylase) that may enable them to interact with their host. Aerotolerance was recorded in *Akkermansia* and *Cloacibacillus* and was also common among representatives of *Bacteroidetes*. On the other hand, *Elusimicrobium* and the majority of *Clostridiales* were highly sensitive to air exposure despite their potential for spore formation.

**Conclusions:**

Major gut microbiota members utilise different strategies for gut colonisation. High oxygen sensitivity of *Firmicutes* may explain their commonly reported decrease after oxidative burst during gut inflammation.

**Electronic supplementary material:**

The online version of this article (10.1186/s12864-018-4959-4) contains supplementary material, which is available to authorized users.

## Background

Characterisation of gut microbiota is nowadays relatively simple due to recent advances in next generation sequencing. However, though DNA sequencing is useful for monitoring dynamic changes in microbiota composition, it does not enable the understanding of biological functions of individual microbiota members. Shotgun sequencing of total DNA or RNA/cDNA sequencing can partly indicate the metabolic potential of microbial communities but is limited in addressing biological functions of individual microbiota members [1–3]. Even in the cases of analysis of microbial communities with low complexity when long DNA contigs can be assembled and associated with a particular bacterium, such approaches do not allow for subsequent experimental verification of observed data due to the unavailability of pure bacterial cultures. Isolation of gut anaerobes in pure cultures is therefore the best way to examine the characteristics of individual microbiota members experimentally [4].

Isolation of bacterial gut microbiota members in pure cultures is usually an issue since the majority of bacterial isolates colonising the intestinal tract are strict anaerobes. Although it is commonly reported that between 10 and 50% of bacterial species colonising the intestinal tract can be grown in vitro, recent studies proposed that up to 90% of major gut colonisers may be grown in vitro if multiple culture conditions are tested [5, 6]. Despite this, the isolation of a particular gut anaerobe may remain an issue since even the most abundant microbiota members at species level only rarely form more than 1% of the total population [7, 8]. This means that the desired bacterial species may be represented by a single colony growing on an agar plate together with hundreds or thousands of others and the likelihood of picking up the particular species is indeed rather low [6].

Chickens represent a specific case for studies focused on host - microbiota interactions. Chickens in commercial production are hatched in a clean environment of hatcheries without any contact with adult hens and their colonisation is dependent on environmental sources. Moreover, newly hatched chickens can be colonised by microbiota of wide a range of compositions [7] and colonisation of the intestinal tract of commercially hatched chickens may therefore differ from the colonisation of intestinal tract of chickens which would hatch in a nest. Perhaps not surprisingly, commercially hatched chickens are highly sensitive to colonisation with different pathogens, e.g. *Salmonella*, nevertheless, their resistance can be increased by providing them with a complex microbiota of adult hens [9, 10].

In our recent studies, we characterised the development of gut microbiota in commercially hatched chickens throughout their whole life [8], identified proteins expressed by the main microbiota members [7] and verified that gut microbiota may protect chickens against *Salmonella* Enteritidis infection [9]. However, which bacterial species are the protective ones is not known and more detailed knowledge of individual microbiota members is needed. One way forward is to obtain well-characterised pure cultures of gut anaerobes [11]. In this study we therefore cultured hundreds of isolates of anaerobes from chicken caecum and sequenced more than 200 of them. Analysis of their genomic sequences showed that we collected isolates from 7 different phyla. The aims of the subsequent comparisons focused mainly on the representatives from phyla *Bacteroidetes* and *Firmicutes* was to reveal differences in (poly)saccharide utilisation, propionate and butyrate fermentation and interactions with the host including motility or the ability to degrade host derived proteins.

## Results

**Fig. 1:**
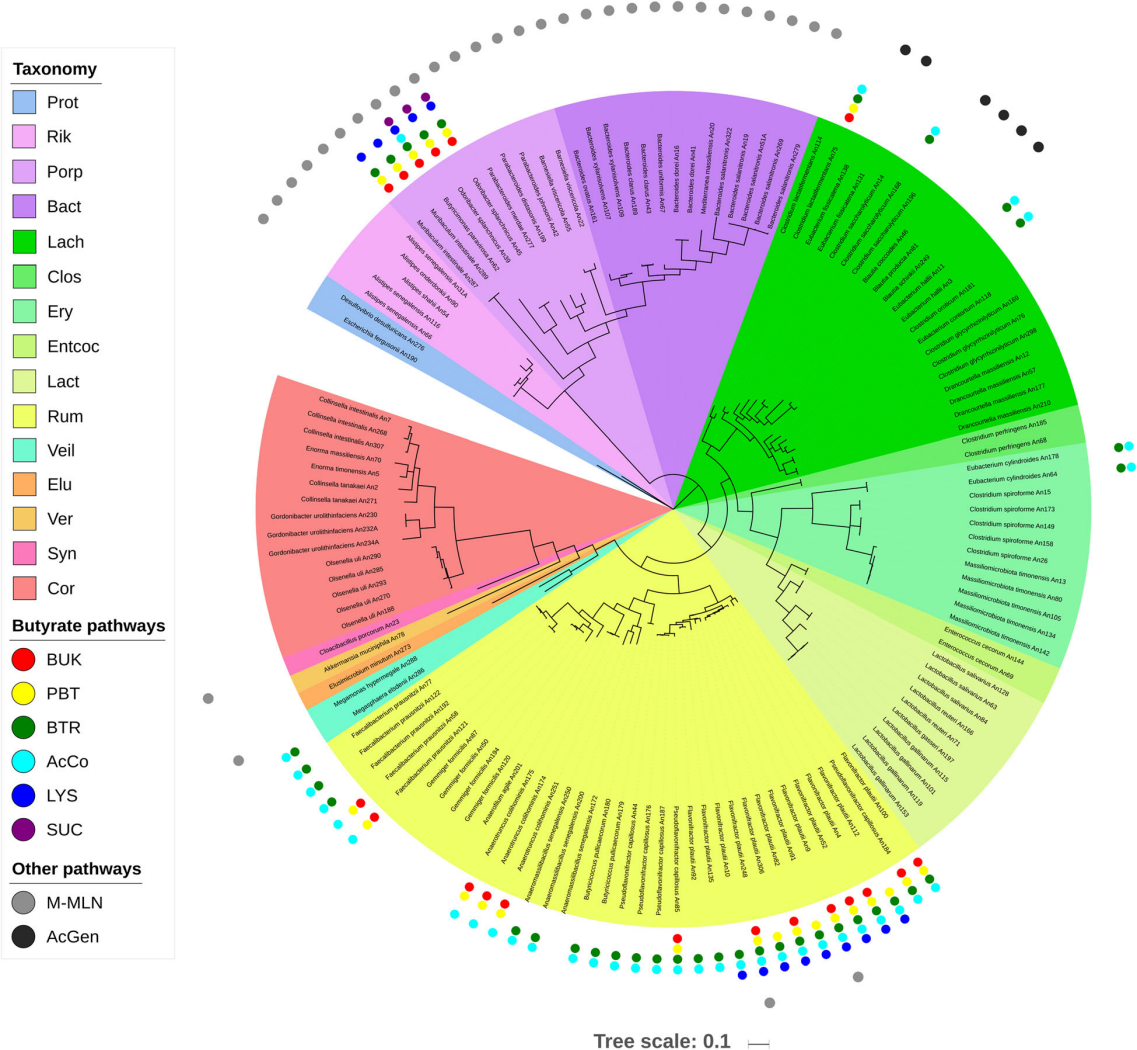
Phylogenetic tree with selected functional properties of 133 sequenced isolates obtained from chicken caecum based on the Bayesian analysis of the full-length sequences of 16S rRNA genes. Families within the phylum *Firmicutes* are shown in light blue, green and yellow. Families within the phylum *Bacteroidetes* are shown in shades of purple. *Actinobacteria* (family *Coriobacteriaceae* – Cor) are highlighted in red. In the cases when only one or two isolates were sequenced per phylum, these isolates are described by phylum name – Prot – *Proteobacteria*, Elu – *Elusimicrobia*, Ver – *Verrucomicrobia*, Syn – *Synergistetes*. In the remaining cases, branches with different families are highlighted with different colors. Rik – *Rikenellaceae*, Porp - *Porphyromonadaceae*, Bact – *Bacteroidaceae*, Lach – *Lachnospiraceae*, Clos - *Clostridiaceae*, Ery – *Erysipelotrichaceae*, Entcoc – *Enterococcaceae*, Lact – *Lactobacillaceae*, Rum – *Ruminococcaceae*, Veil – *Veillonellaceae*. BUK – butyrate kinase, BPT – phosphate butyryltransferase, BTR – butyryl-CoA transferase, AcCo – acetyl CoA pathway, LYS – lysine fermentation pathway, SUC – succinate fermentation pathway, M-MLN presence of methylmalonyl mutase, epimerase and decarboxylase required for conversion of succinate to methyl-malonyl CoA and propionate. AcGen – presence of genes required for reductive acetogenesis

Altogether 204 isolates were obtained in pure culture and sequenced. Since in several cases we sequenced isolates exhibiting higher than 99% sequence similarity between their genomes, the final number of different isolates decreased to 133 (Table 1, Fig. 1). The lowest sequencing coverage was 43 fold for *Drancourtella massiliensis* An12, the highest 1526 fold for *Lactobacillus gasseri* An197, and median coverage over all sequenced isolates was 312 fold (see Additional file 1 for all details). The isolates belonged to 7 different phyla – *Firmicutes* (84 isolates), *Bacteroidetes* (29 isolates), *Actinobacteria* (15 isolates), *Proteobacteria* (1 isolate each of *Escherichia* and *Desulfovibrio*), *Verrucomicrobia* (1 isolate of *Akkermansia*), *Elusimicrobia* (1 isolate of *Elusimicrobium*) and *Synergistetes* (1 isolate of *Cloacibacillus*). The similarity of whole sequences of 16S rRNA to the to the most homologous GenBank entries was lower than 94% for 15 isolates. Considering taxonomic recommendations [12], these isolates represent candidates for new genera and two *Muribaculum*-like isolates might belong to a novel bacterial family or even an order. Alternative analysis based on alignment of RpoB amino acid sequences [13] yielded similar clustering of individual isolates as that achieved by 16S rRNA comparison (Additional file 2).

**Table 1:** List of strains isolated, sequenced and analysed in this study

Phylum	Family	Species	ID	rRNA % sim.^a^	genome (bp)	GC (%)
Actinobacteria	Coriobacteriaceae	Collinsella intestinalis	An268	94	2,375,164	65.28
Actinobacteria	Coriobacteriaceae	Collinsella intestinalis	An7	95	2,368,937	65.33
Actinobacteria	Coriobacteriaceae	Collinsella intestinalis	An307	96	2,218,890	62.95
Actinobacteria	Coriobacteriaceae	Collinsella tanakaei	An271	96	2,790,023	64.34
Actinobacteria	Coriobacteriaceae	Collinsella tanakaei	An2	94	2,542,639	62.39
Actinobacteria	Coriobacteriaceae	Enorma massiliensis	An70	99	2,374,054	62.09
Actinobacteria	Coriobacteriaceae	Enorma timonensis	An5	96	2,299,978	65.99
Actinobacteria	Coriobacteriaceae	Gordonibacter urolithinfaciens	An234A	99	3,542,488	65.99
Actinobacteria	Coriobacteriaceae	Gordonibacter urolithinfaciens	An232A	94	3,457,314	65.16
Actinobacteria	Coriobacteriaceae	Gordonibacter urolithinfaciens	An230	94	3,885,330	64.16
Actinobacteria	Coriobacteriaceae	Olsenella uli	An188	96	2,138,506	68.68
Actinobacteria	Coriobacteriaceae	Olsenella uli	An290	96	2,158,343	68.09
Actinobacteria	Coriobacteriaceae	Olsenella uli	An285	96	2,413,556	66.98
Actinobacteria	Coriobacteriaceae	Olsenella uli	An293	96	2,318,914	67.87
Actinobacteria	Coriobacteriaceae	Olsenella uli	An270	96	2,014,154	67.55
Bacteroidetes	Bacteroidaceae	Bacteroides clarus	An43	99	4,226,284	45.22
Bacteroidetes	Bacteroidaceae	Bacteroides clarus	An189	99	4,166,078	45.36
Bacteroidetes	Bacteroidaceae	Bacteroides dorei	An16	99	5,376,103	41.79
Bacteroidetes	Bacteroidaceae	Bacteroides dorei	An41	99	5,463,463	42.00
Bacteroidetes	Bacteroidaceae	Bacteroides ovatus	An161	99	6,700,861	42.08
Bacteroidetes	Bacteroidaceae	Bacteroides salanitronis	An322	92	3,449,415	44.84
Bacteroidetes	Bacteroidaceae	Bacteroides uniformis	An67	99	4,590,834	46.36
Bacteroidetes	Bacteroidaceae	Bacteroides xylanisolvens	An109	99	5,713,108	41.57
Bacteroidetes	Bacteroidaceae	Bacteroides xylanisolvens	An107	99	5,745,201	41.89
Bacteroidetes	Bacteroidaceae	Mediterranea massiliensis	An20	91	3,968,548	49.41
Bacteroidetes	Porphyromonadaceae	Barnesiella viscericola	An22	98	3,264,150	53.04
Bacteroidetes	Porphyromonadaceae	Barnesiella viscericola	An55	98	3,040,531	51.37
Bacteroidetes	Porphyromonadaceae	Butyricimonas paravirosa	An62	98	5,176,855	42.63
Bacteroidetes	Porphyromonadaceae	Muribaculum intestinale	An289	83	2,336,263	49.09
Bacteroidetes	Porphyromonadaceae	Muribaculum intestinale	An287	83	2,349,712	49.04
Bacteroidetes	Porphyromonadaceae	Odoribacter splanchnicus	An45	99	4,610,398	43.75
Bacteroidetes	Porphyromonadaceae	Odoribacter splanchnicus	An39	99	4,722,515	43.48
Bacteroidetes	Porphyromonadaceae	Parabacteroides distasonis	An199	99	5,145,110	45.11
Bacteroidetes	Porphyromonadaceae	Parabacteroides johnsonii	An42	99	4,430,164	45.03
Bacteroidetes	Porphyromonadaceae	Parabacteroides merdae	An277	92	3,709,857	46.69
Bacteroidetes	Rikenellaceae	Alistipes onderdonkii	An90	100	3,488,443	58.52
Bacteroidetes	Rikenellaceae	Alistipes senegalensis	An31A	97	2,626,508	61.85
Bacteroidetes	Rikenellaceae	Alistipes senegalensis	An116	97	3,264,205	58.57
Bacteroidetes	Rikenellaceae	Alistipes senegalensis	An66	97	3,064,564	59.37
Bacteroidetes	Rikenellaceae	Alistipes shahii	An54	96	3,272,633	58.38
Bacteroidetes	unclassified_"Bacteroidales”	Bacteroides salanitronis	An269	92	4,466,522	45.87
Bacteroidetes	unclassified_"Bacteroidales”	Bacteroides salanitronis	An279	92	3,976,735	45.82
Bacteroidetes	unclassified_"Bacteroidales”	Bacteroides salanitronis	An19	92	4,779,606	45.88
Bacteroidetes	unclassified_"Bacteroidales”	Bacteroides salanitronis	An51A	92	4,415,476	45.79
Firmicutes	Enterococcaceae	Enterococcus cecorum	An69	100	2,010,800	36.66
Firmicutes	Enterococcaceae	Enterococcus cecorum	An144	100	2,521,030	36.10
Firmicutes	Lactobacillaceae	Lactobacillus gallinarum	An119	99	2,068,702	36.50
Firmicutes	Lactobacillaceae	Lactobacillus gallinarum	An153	99	2,042,196	36.47
Firmicutes	Lactobacillaceae	Lactobacillus gallinarum	An115	99	2,039,377	36.52
Firmicutes	Lactobacillaceae	Lactobacillus gallinarum	An101	99	2,051,080	36.54
Firmicutes	Lactobacillaceae	Lactobacillus gasseri	An197	99	1,786,561	34.60
Firmicutes	Lactobacillaceae	Lactobacillus reuteri	An71	99	2,330,171	38.47
Firmicutes	Lactobacillaceae	Lactobacillus reuteri	An166	99	2,293,009	38.50
Firmicutes	Lactobacillaceae	Lactobacillus salivarius	An63	99	1,826,390	32.76
Firmicutes	Lactobacillaceae	Lactobacillus salivarius	An84	99	2,115,320	32.74
Firmicutes	Lactobacillaceae	Lactobacillus salivarius	An128	99	1,900,005	32.75
Firmicutes	Erysipelotrichaceae	[Clostridium] spiroforme	An158	99	2,749,214	28.90
Firmicutes	Erysipelotrichaceae	[Clostridium] spiroforme	An15	94	3,043,058	30.24
Firmicutes	Erysipelotrichaceae	[Clostridium] spiroforme	An26	99	2,686,825	28.58
Firmicutes	Erysipelotrichaceae	[Clostridium] spiroforme	An149	99	2,786,011	28.86
Firmicutes	Erysipelotrichaceae	[Clostridium] spiroforme	An173	94	2,963,161	30.12
Firmicutes	Erysipelotrichaceae	[Eubacterium] cylindroides	An64	99	1,822,768	34.80
Firmicutes	Erysipelotrichaceae	[Eubacterium] cylindroides	An178	99	1,979,688	34.75
Firmicutes	Erysipelotrichaceae	Massiliomicrobiota timonensis	An13	100	2,808,053	31.46
Firmicutes	Erysipelotrichaceae	Massiliomicrobiota timonensis	An142	98	2,805,108	31.51
Firmicutes	Erysipelotrichaceae	Massiliomicrobiota timonensis	An134	98	2,583,105	31.58
Firmicutes	Erysipelotrichaceae	Massiliomicrobiota timonensis	An105	98	2,657,304	31.69
Firmicutes	Erysipelotrichaceae	Massiliomicrobiota timonensis	An80	98	2,632,118	31.88
Firmicutes	Clostridiaceae 1	*Clostridium perfringens*	An68	99	3,279,733	28.07
Firmicutes	Clostridiaceae 1	Clostridium perfringens	An185	99	3,267,175	28.06
Firmicutes	Lachnospiraceae	[Clostridium] glycyrrhizinilyticum	An169	96	4,874,615	49.44
Firmicutes	Lachnospiraceae	[Clostridium] glycyrrhizinilyticum	An298	96	3,190,622	46.76
Firmicutes	Lachnospiraceae	[Clostridium] glycyrrhizinilyticum	An76	96	3,392,322	50.34
Firmicutes	Lachnospiraceae	[Clostridium] lactatifermentans	An114	95	2,765,027	31.21
Firmicutes	Lachnospiraceae	[Clostridium] lactatifermentans	An75	99	3,241,294	44.78
Firmicutes	Lachnospiraceae	[Clostridium] oroticum	An181	95	2,778,317	43.19
Firmicutes	Lachnospiraceae	[Clostridium] saccharolyticum	An14	95	4,319,160	54.42
Firmicutes	Lachnospiraceae	[Clostridium] saccharolyticum	An168	99	3,501,825	50.31
Firmicutes	Lachnospiraceae	[Clostridium] saccharolyticum	An196	93	3,261,385	50.05
Firmicutes	Lachnospiraceae	[Eubacterium] contortum	An118	95	3,480,620	52.25
Firmicutes	Lachnospiraceae	[Eubacterium] fissicatena	An131	94	3,529,099	50.87
Firmicutes	Lachnospiraceae	[Eubacterium] fissicatena	An138	94	3,702,317	50.44
Firmicutes	Lachnospiraceae	[Eubacterium] hallii	An3	95	4,260,545	46.43
Firmicutes	Lachnospiraceae	[Eubacterium] hallii	An11	95	3,929,296	46.78
Firmicutes	Lachnospiraceae	Blautia coccoides	An46	94	3,844,348	44.62
Firmicutes	Lachnospiraceae	Blautia producta	An81	95	4,340,741	44.56
Firmicutes	Lachnospiraceae	Blautia schinkii	An249	93	3,969,115	45.08
Firmicutes	Lachnospiraceae	Drancourtella massiliensis	An210	95	3,049,918	45.07
Firmicutes	Lachnospiraceae	Drancourtella massiliensis	An177	95	2,986,142	44.76
Firmicutes	Lachnospiraceae	Drancourtella massiliensis	An57	95	3,307,621	45.72
Firmicutes	Lachnospiraceae	Drancourtella massiliensis	An12	96	3,637,323	46.18
Firmicutes	Ruminococcaceae	Anaerofilum agile	An201	91	3,232,216	60.34
Firmicutes	Ruminococcaceae	Anaeromassilibacillus senegalensis	An200	94	3,738,663	54.30
Firmicutes	Ruminococcaceae	Anaeromassilibacillus senegalensis	An250	97	3,582,839	53.28
Firmicutes	Ruminococcaceae	Anaeromassilibacillus senegalensis	An172	92	2,820,032	41.40
Firmicutes	Ruminococcaceae	Anaerotruncus colihominis	An174	99	4,104,028	53.46
Firmicutes	Ruminococcaceae	Anaerotruncus colihominis	An175	99	4,098,164	53.45
Firmicutes	Ruminococcaceae	Anaerotruncus colihominis	An251	99	3,446,606	54.60
Firmicutes	Ruminococcaceae	Butyricicoccus pullicaecorum	An179	99	3,474,625	53.61
Firmicutes	Ruminococcaceae	Butyricicoccus pullicaecorum	An180	99	3,016,034	54.43
Firmicutes	Ruminococcaceae	Faecalibacterium prausnitzii	An121	95	2,728,163	61.06
Firmicutes	Ruminococcaceae	Faecalibacterium prausnitzii	An77	96	3,092,450	59.48
Firmicutes	Ruminococcaceae	Faecalibacterium prausnitzii	An192	97	3,520,066	58.80
Firmicutes	Ruminococcaceae	Faecalibacterium prausnitzii	An122	96	3,272,200	59.47
Firmicutes	Ruminococcaceae	Faecalibacterium prausnitzii	An58	96	2,956,350	60.63
Firmicutes	Ruminococcaceae	Flavonifractor plautii	An91	97	3,629,120	57.60
Firmicutes	Ruminococcaceae	Flavonifractor plautii	An52	97	2,845,789	59.13
Firmicutes	Ruminococcaceae	Flavonifractor plautii	An92	98	3,498,453	62.13
Firmicutes	Ruminococcaceae	Flavonifractor plautii	An112	97	2,967,986	59.27
Firmicutes	Ruminococcaceae	Flavonifractor plautii	An135	98	3,907,586	61.45
Firmicutes	Ruminococcaceae	Flavonifractor plautii	An82	97	3,683,590	58.85
Firmicutes	Ruminococcaceae	Flavonifractor plautii	An248	100	3,776,725	60.99
Firmicutes	Ruminococcaceae	Flavonifractor plautii	An4	97	3,239,670	58.22
Firmicutes	Ruminococcaceae	Flavonifractor plautii	An9	97	3,357,728	58.20
Firmicutes	Ruminococcaceae	Flavonifractor plautii	An100	95	3,054,748	57.15
Firmicutes	Ruminococcaceae	Flavonifractor plautii	An306	98	3,967,264	59.00
Firmicutes	Ruminococcaceae	Flavonifractor plautii	An10	96	3,867,419	61.74
Firmicutes	Ruminococcaceae	Gemmiger formicilis	An50	94	3,597,344	58.67
Firmicutes	Ruminococcaceae	Gemmiger formicilis	An194	95	3,080,663	59.30
Firmicutes	Ruminococcaceae	Gemmiger formicilis	An87	94	3,355,244	58.58
Firmicutes	Ruminococcaceae	Gemmiger formicilis	An120	95	3,406,814	60.15
Firmicutes	Ruminococcaceae	Pseudoflavonifractor capillosus	An176	96	2,574,287	58.19
Firmicutes	Ruminococcaceae	Pseudoflavonifractor capillosus	An187	96	2,623,188	57.97
Firmicutes	Ruminococcaceae	Pseudoflavonifractor capillosus	An44	96	2,764,660	57.41
Firmicutes	Ruminococcaceae	Pseudoflavonifractor capillosus	An85	96	3,018,026	56.20
Firmicutes	Ruminococcaceae	Pseudoflavonifractor capillosus	An184	96	3,617,258	59.87
Firmicutes	Veillonellaceae	Megamonas hypermegale	An288	99	2,165,576	33.61
Firmicutes	Veillonellaceae	Megasphaera elsdenii	An286	95	2,396,433	53.29
Proteobacteria	Desulfovibrionaceae	*Desulfovibrio desulfuricans*	An276	92	3,230,576	55.37
Proteobacteria	Enterobacteriaceae	Escherichia fergusonii	An190	99	5,352,565	50.48
Synergistetes	Synergistaceae	Cloacibacillus porcorum	An23	93	2,902,045	57.89
Verrucomicrobia	Verrucomicrobiaceae	Akkermansia muciniphila	An78	100	2,734,062	55.68
Elusimicrobia	Elusimicrobiaceae	Elusimicrobium minutum	An273	94	1,505,722	51.73

^a^Percent similarity along the whole 16S rRNA sequence to the GenBank entry with the highest similarity

When we compared the sequence of 16S rRNA genes of all 133 isolates with the operational taxonomic units (OTUs) combined from our previous studies [7, 8], rather unexpectedly, 7 isolates were excluded from the analysis by QIIME at the chimera removal step. Three isolates formed OTUs which we did not detect among the microbiota of the two studies. The rest of the isolates were assigned into particular OTUs. Nineteen isolates were assigned to 11 OTUs from the 100 most frequent OTUs, and out of the 1000 most common OTUs, we obtained 76 isolates belonging to 42 OTUs. Exact ranking of each individual isolate among the most frequent OTUs present in the chicken caecum can be found in Additional file 1.

Genome sizes ranged from 1.51 Mb in *Elusimicrobium* to 6.70 Mb in *Bacteroides ovatus*. Larger genomes were usually recorded in isolates from phylum *Bacteroidetes* (Additional file 1). *Actinobacteria* possessed small genomes ranging from 2.1 to 2.5 Mb. Genomes of *Firmicutes* ranged mostly from 3 to 4 Mb although genomes of *Enterococcus*, *[Eubacterium] cylindroides* and *Lactobacillus* were among the smallest ones with genome sizes around 2 Mb. Genomic GC content ranged from 28.0 to 62.1% in *Firmicutes*, from 41.6 to 61.9% in *Bacteroidetes* and from 62.1 to 68.7% in *Actinobacteria*. GC content of individual isolates belonging to the remaining 4 phyla ranged from 50.5 to 57.9% (Additional file 1).

### Whole genome comparison

**Fig. 2:**
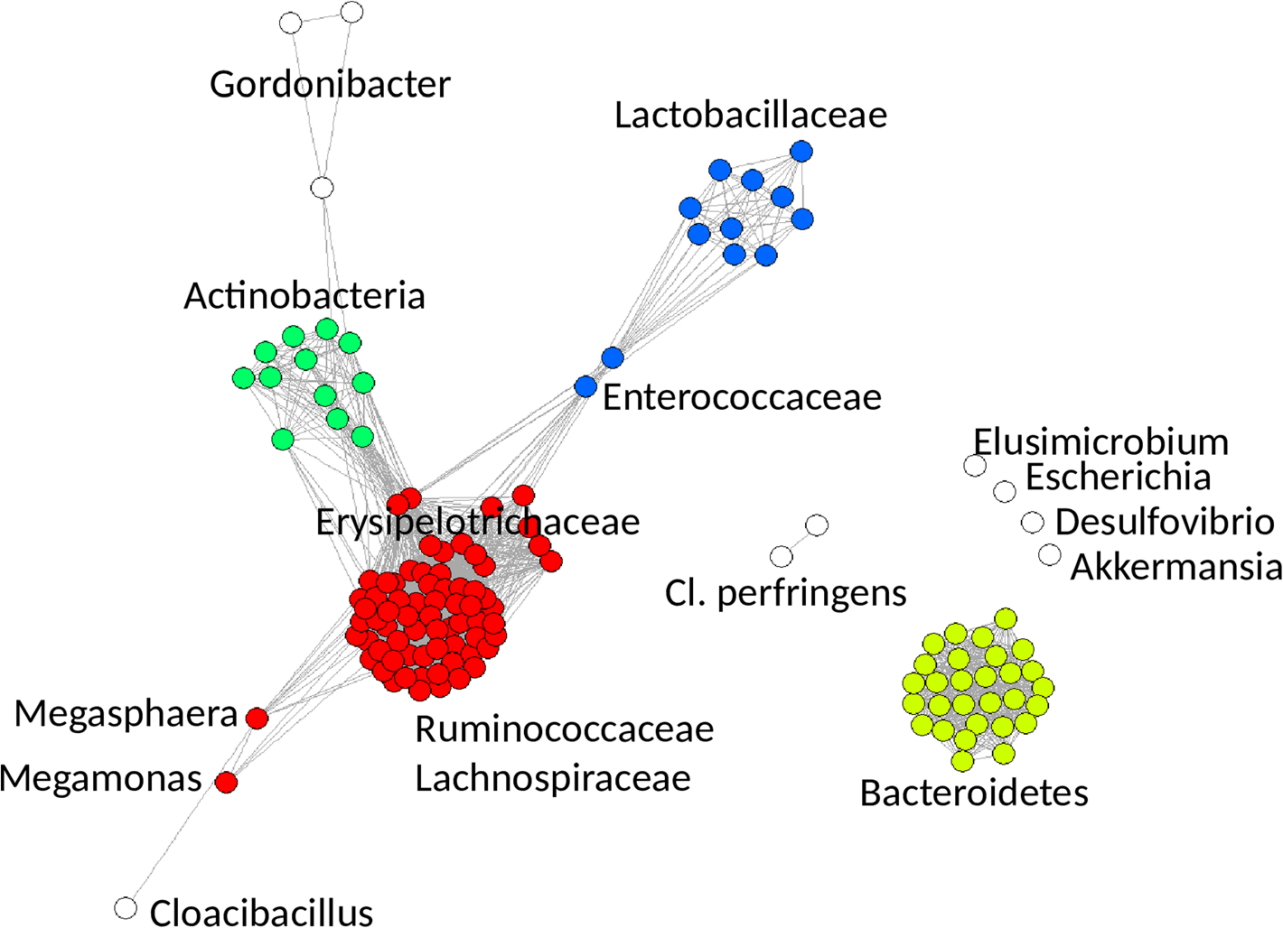
Network analysis of isolates from chicken caecum based on their gene composition. *Firmicutes* split into families *Lactobacillaceae*, *Enterococcaceae*, *Veillonellaceae* (genera *Megasphaera* and *Megamonas*) and the order *Clostridiales* except for *Clostridium perfringens*. Separation of *Erysipelotrichaceae* from *Lachnospiraceae* and *Ruminococcaceae* (all belonging to order *Clostridiales*) was observed. All *Bacteroidetes* formed a separated cluster showing similar genetic composition in all isolates. The majority of *Actinobacteria* formed a single cluster except for the representatives of the genus *Gordonibacter* indicating that genetic composition of these isolates differed from the rest of *Actinobacteria*

Network analysis based on the correlation of individual gene counts in individual isolates confirmed similarities observed by 16S rRNA gene alignment. Individual isolates from phyla *Proteobacteria*, *Verrucomicrobia*, *Elusimicrobia* and *Synergistetes* formed disconnected vertices of the network (Fig. 2). *Firmicutes* split into families *Lactobacillaceae*, *Enterococcaceae*, *Veillonellaceae* (genera *Megasphaera* and *Megamonas*) and order *Clostridiales* (families *Erysipelotrichaceae*, *Ruminococcaceae* and *Lachnospiraceae*) except for *Clostridium perfringens*. Members of the family *Erysipelotrichaceae* formed a slightly eccentric cluster at the periphery of other isolates from the order *Clostridiales* indicating their slightly different coding capacity when compared to isolates belonging to families *Lachnospiraceae* and *Ruminococcaceae*. All *Bacteroidetes* formed a disconnected network cluster showing similar genetic composition in all isolates. The majority of *Actinobacteria* formed a single network cluster except for the representatives of genus *Gordonibacter* showing that the genome of this genus differed from the rest of *Actinobacteria* sequenced in this study.

### Basic biological processes

**Fig. 3:**
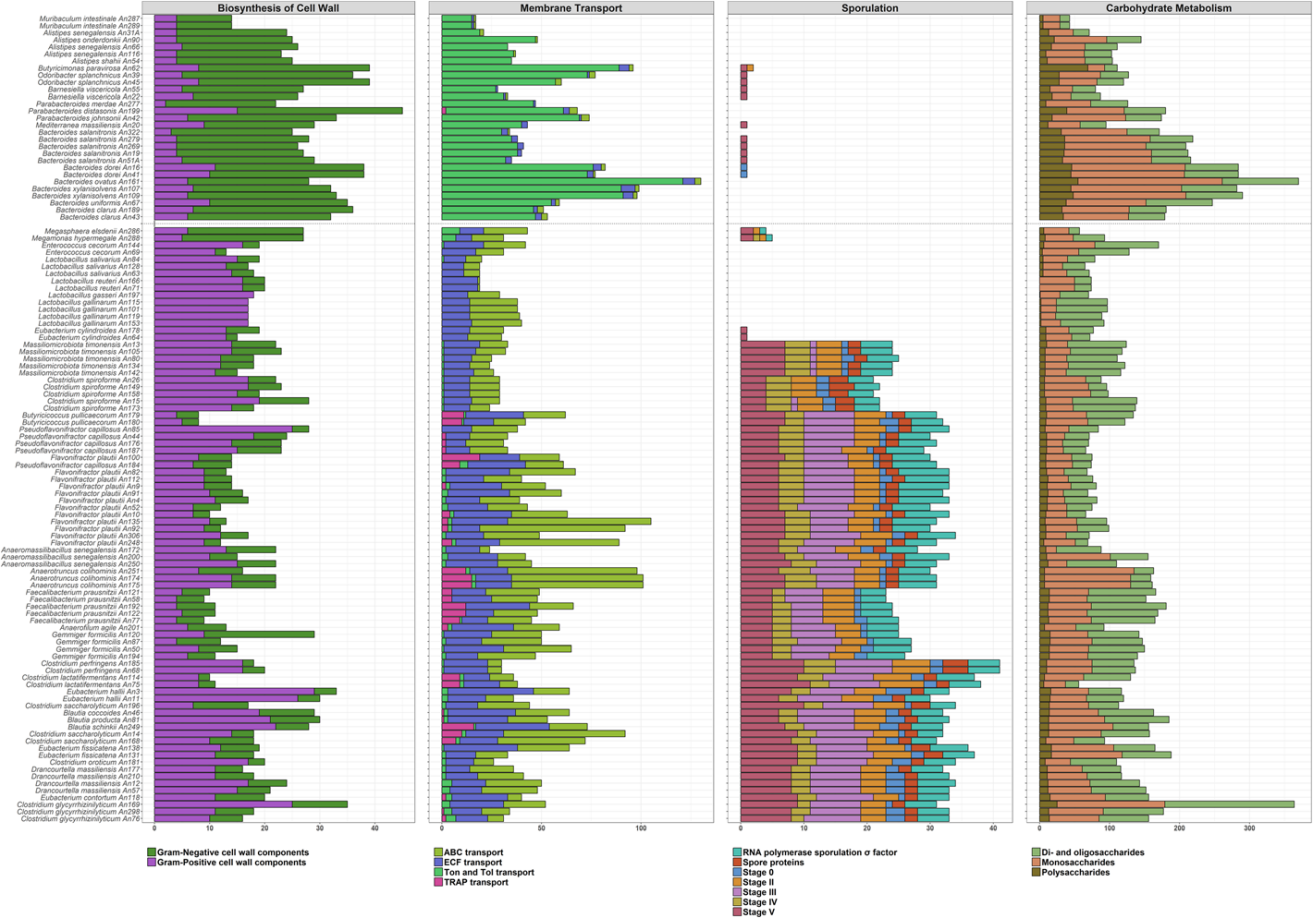
Distribution of genes in selected categories among representatives of major gut colonisers belonging to phyla *Bacteroidetes* and *Firmicutes.* X axes indicate the numbers of genes in a given category per genome. For a scalable figure see Additional file 5 online

**Fig. 4:**
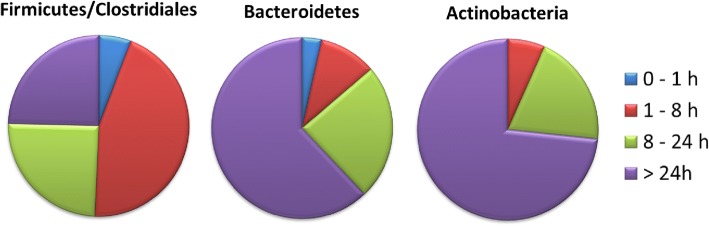
Sensitivity of gut anaerobes to air exposure. Most of isolates from the order *Clostridiales*, phylum *Firmicutes* (*n* = 69) died during 1–8 long hour exposure to the air. On the other hand, majority of the tested representatives of *Bacteroidetes* (*n* = 29) and *Actinobacteria* (*n* = 15) survived 24-h-long exposure to air. For full information on survival of individual isolates see Additional file 3 online

Since gut microbiota is formed mainly by representatives of phyla *Bacteroidetes* and *Firmicutes*, we specifically focused on the comparison of genomes of isolates belonging to these two phyla. Representatives belonging to phylum *Bacteroidetes* (Gram negative bacteria) coded for proteins required for the biosynthesis of a Gram negative cell wall while representatives of phylum *Firmicutes* (Gram positive bacteria) coded for proteins required for the biosynthesis of a Gram positive cell wall. However, *Megamonas* and *Megasphaera* (family *Veilonellaceae*, phylum *Firmicutes,* i.e. Gram positive bacteria) harboured genes required for the synthesis of Gram negative cell wall type (Fig. 3). *Bacteroidetes* and *Firmicutes* differed in their mode of transport across the cell wall and cytoplasmic membrane. In *Bacteroidetes*, genes belonging to Ton and Tol transport systems were the most frequent whilst *Firmicutes* encoded ABC transporters, ECF class transporters and TRAP transporters (Fig. 3). Genes enabling sporulation were specific to Gram positive *Firmicutes* except for *Lactobacillaceae*, *Enterococcaceae*, *Veilonellaceae* and two [*Eubacterium*] *cylindroides* isolates (Fig. 3). However, there were differences in the composition and distribution of genes necessary for spore formation among the isolates with sporulation potential. Members of the family *Erysipelotrichaceae* (*Massiliomicrobiota* sp. and [*Clostridium*] *spiroforme*) did not code for stage III sporulation proteins and *Faecalibacterium*, *Anaerofilum* and *Gemmiger* (family *Ruminococcaceae*) did not code for spore proteins though the rest of the genes required for sporulation were present in their genomes (Fig. 3). None of the *Bacteroidetes* isolates encoded proteins required for sporulation and their oxygen tolerance could be dependent on *batABCDE* operon (*Bacteroides* aerotolerance). *batABE* genes were present in all representatives of *Bacteroidetes* and *batCD* were present in all *Bacteroidetes* except for two *Muribaculum* isolates. When we examined the survival rate of the anaerobes after exposure to aerobic conditions experimentally, the most sensitive isolates were single isolates of *Muribaculum intestinale* (phylum *Bacteroidetes*), [*Clostridium*] *glycyrrhizinilyticum*, [*Clostridium*] *saccharolyticum*, *Anaeromassilibacillus senegalensis* and *Flavonifractor plautii* (all phylum *Firmicutes*) which did not survive an hour long exposure to the air. Bacteria which did not survive 8 or 24-h-long exposure to the air belonged mainly to the order *Clostridiales*. Within *Clostridiales*, 45% of the tested isolates did not survive 8-h long exposure to the air, and an additional 25% died between 8 to 24-h exposure. Only 25% of tested isolates from the order *Clostridiales* survived 24-h air exposure. On the other hand, representatives of *Bacteroidetes* and *Actinobacteria* were usually tolerant to sudden air exposure since 62 and 73% of tested isolates survived 24-h air exposure, respectively (Fig. 4 and Additional file 3). *Bacteroides* sp. encoded a high number of proteins involved in polysaccharide and monosacharide metabolism while the number of genes required for metabolism of di- and oligosaccharides was similar in both *Bacteroidetes* and *Firmicutes* (Fig. 3).

### Production of butyrate, propionate and acetogenesis

Short chain fatty acids and butyrate in particular, are acknowledged as important metabolites of bacterial origin [14, 15]. All genes required for butyrate production from pyruvate and acetyl-CoA were present in the genomes of *Ruminococcaceae* (genera *Butyricicoccus*, *Pseudoflavonifractor*, *Flavonifractor*, *Anaeromassilibacillus*, *Anaerotruncus* and *Faecalibacterium*) (Fig. 1). In addition, this pathway was also present in *Megasphaera elsdenii*, [*Eubacterium*] *cylindroides*, [*Clostridium*] *lactatifermentans*, [*Clostridium*] *saccharolyticum* and [*Eubacterium*] *hallii*, the latter three belonging to the family *Lachnospiraceae* (Fig. 1). *Butyricimonas paravirosa* was the only isolate from phylum *Bacteroidetes* coding for all genes required for butyrate production from pyruvate and acetyl-CoA. Genes coding for enzymes of complete lysine fermentation pathway leading to butyrate production were present in three genera of the phylum *Bacteroidetes* (*Muribaculum*, *Butyricimonas* and *Odoribacter*) and the majority of *Flavonifractor* isolates belonging to the phylum *Firmicutes*. *Butyricimonas* and *Odoribacter* also encoded the whole pathway required for the conversion of succinate and 4-hydroxybutyrate into butyrate (Fig. 1). Terminal steps in butyrate production were dependent on transferases transferring CoA moiety from butyryl-CoA to acetate, acetoacetate or 4-hydroxybutyrate, or phosphate butyryltransferase (PBT) and butyrate kinase (BUK). Surprisingly, we did not find butyrate kinase in all isolates using the PBT - BUK pathway for butyrate release from butyryl-CoA. In such a case, butyrate-phosphate may serve for substrate phosphorylation in enzymatic reactions similar to acetate-phosphate.

Propionate production via a succinate-methylmalonate pathway was characteristic of *Bacteroidetes* (Fig. 1) as genes for methylmalonyl-CoA mutase, epimerase and decarboxylase were detected in genomes of all isolates from this phylum. This pathway was quite rare in *Firmicutes* since methylmalonyl-CoA mutase, epimerase and decarboxylase were encoded only by *Megamonas* and two *Flavonifractor* isolates (Fig. 1).

Fermentation of carbohydrates results in the production of H_2_ which has to be removed from the community since its increased concentration suppresses glycolysis [16–18]. H_2_ can be removed by methanogens, acetogens or sulphate reducing bacteria. We did not isolate a single methanogen. *Desulfovibrio* (phylum *Proteobacteria*) encoded key genes for sulphate reduction to H_2_S (adenylylsulphate reductase, sulphate adenylyltransferase, dissimilatory sulphite reductase and sulphite reduction-associated complex DsrMKJOP). Potential for H_2_ removal by acetogenesis was recorded in [*Clostridium*] *saccharolyticum*, [*Eubacterium*] *fissicatena* and all *Blautia* isolates (*B*. *coccoides*, *B. producta*, *B*. *schinkii*) since all these bacteria encoded corrinoid iron-sulfur acetyl-CoA synthase and 5-methyltetrahydrofolate methyltransferase (Fig. 1).

### Genes encoding proteins mediating interactions with the host

**Fig. 5:**
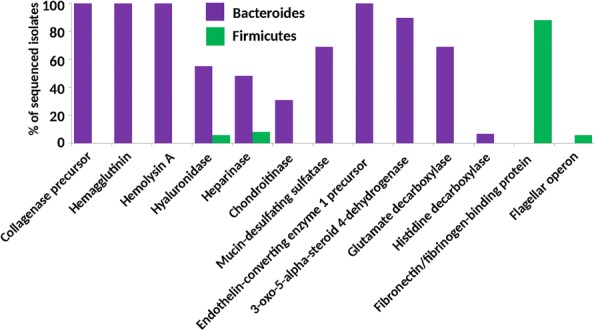
Distribution of genes encoding proteins mediating interactions of *Bacteroidetes* and *Firmicutes* with the host. The presence of genes which may allow for interactions with chicken host structures was determined in each isolate belonging to these two phyla and expressed as a percentage of positive isolates out of all the sequenced *Bacteroidetes* (29 isolates in a total) and *Firmicutes* (84 isolates in a total)

Since gut microbiota may interact with its host, finally we searched for the presence of genes which may facilitate such interactions (Additional file 4). Genes encoding collagenase precursor, hemagglutinin or hemolysin A were present in all 29 *Bacteroidetes* isolates but not in *Firmicutes* (Fig. 5). Hyaluronidase was present in 16 isolates from the phylum *Bacteroidetes* and five *Firmicutes*. Different heparinases were detected in 14 isolates from the phylum *Bacteroidetes* and seven representatives of *Firmicutes* – out of these all five *Faecalibacterium* isolates encoded heparinase II/III-like and outside this genus, heparinases were present only in two isolates from the phylum *Firmicutes* (i.e. *Gemmiger formicilis* and [*Clostridium*] *glycyrrhizinilyticum*). Chondroitinase was present in nine isolates from phylum *Bacteroidetes* (genera *Alistipes* and *Bacteroides*) but in no isolate from *Firmicutes*. Mucin-desulfating sulfatase was present in the genomes of 20 *Bacteroidetes* isolates (mainly in genera *Alistipes*, *Parabacteroides* and *Bacteroides*) but was absent from the genomes of *Firmicutes*. A gene for endothelin-converting enzyme 1 precursor was present in the genomes of all *Bacteroidetes* but not in *Firmicutes*. Except for two *Odoribacter* isolates and *Parabacteroides distatonis*, all the remaining representatives of the phylum *Bacteroidetes* encoded 3-oxo-5-α-steroid 4-dehydrogenase capable of modification of bile or steroid hormones. This gene was not detected in *Firmicutes*. Glutamate decarboxylase catalysing production of γ-aminobutyrate (GABA) and glutamate/GABA antiporter was present in 20 different *Bacteroidetes* isolates but only in two *Firmicutes* and these were both pathogenic *Clostridium perfringens* isolates. Histidine decarboxylase catalysing production of histamine was quite rare and was present only in two *Bacteroides dorei* isolates (Fig. 5). On the other hand, except for all *Lactobacilli*, the gene for fibronectin/fibrinogen-binding protein was present in all isolates from *Firmicutes* but in none representative from the phylum *Bacteroidetes*. Genes for flagellar motility were quite rare and were absent in all *Bacteroidetes* isolates. Complete flagellar operon was present only in two *Flavonifractor* isolates, one strain of *Anaeromassilibacillus senegalensis* and all three isolates of *Anaerotruncus colihominis* (Fig. 5).

## Discussion

In this study we sequenced, annotated and analysed genomes of 133 different anaerobes cultured from the chicken caecum. Several isolates represented species which are available in only a few pure cultures worldwide – a single manuscript reported a pure culture of *Elusimicrobium* [19] and only two papers reported culture of *Cloacibacillus porcorum* [20, 21]. Although such isolates are of clear potential for future experiments, in this report we mainly focused on the comparison of genetic potential of isolates belonging to two main phyla inhabiting the intestinal tract of chickens, *Bacteroidetes* and *Firmicutes* [8, 18].

Whole genome sequencing showed that representatives of *Bacteroidetes* and *Firmicutes* have a genetic potential to follow different strategies of gut colonisation which may explain their coexistence in the intestinal tract. *Bacteroidetes* encoded genes for the biosynthesis of a Gram negative cell wall, Gram positive anaerobes encoded genes for the biosynthesis of a Gram positive cell wall. Only *Megamonas* and *Megasphaera*, though belonging to Gram positive *Firmicutes*, encoded genes for the biosynthesis of Gram negative cell wall type, in agreement with previous report [22]. The Ton/ExbD transport system of *Bacteroidetes* has been identified earlier as highly expressed in vivo [7] and ABC, ECF and TRAP transporters were described as characteristic of *Firmicutes* [23–25]. *Bacteroidetes* were reported to increase in gut microbiota with high fiber content in their diet [26, 27] and to forage on host derived polysaccharides in the absence of fiber [28–30]. Consistent with this, *Bacteroidetes* and the genus *Bacteroides* in particular encoded a high number of genes required for polysaccharide metabolism [28, 30–33]. Since the polysaccharides of feed and host origin consist of various (amino)monosaccharides, *Bacteroidetes* encoded also a wider range of genes required for monosaccharide degradation than *Firmicutes* (Fig. 3).

Butyrate is produced mainly by *Firmicutes*. Carbohydrate fermentation to pyruvate and acetyl-CoA was the most frequent butyrate production pathway, as proposed earlier [34]. Butyrate production was mainly associated with *Ruminococcaceae* and less frequently with *Lachnospiraceae* or *Erysipelotrichaceae* [35]. *Bacteroidetes* are not the main butyrate producers as acetyl-CoA conversion to butyrate was only found in *Butyricimonas*. However, *Bacteroidetes* were capable of butyrate production by alternative pathways, e.g. from 4-hydroxybutyrate as recorded in *Butyricimonas* and *Odoribacter* or by lysine fermentation as recorded in *Muribaculum*, *Butyricimonas* and *Odoribacter*. This is in line with conclusions derived from human microbiota studies [35].

Gut microbiota interacts with the host. The potential of *Firmicutes* to interact with the chicken host seems to be less extensive in comparison to *Bacteroidetes*. Except for *Lactobacilli*, *Firmicutes* isolates encoded fibronectin/fibrinogen-binding protein [36, 37]. Interestingly, we detected chicken fibrinogen-domain containing proteins as tightly associated with gut microbiota [38]. Such proteins aggregate bacteria [39, 40] and enable association of different gut microbiota members, based on current results, preferentially those belonging to the phylum *Firmicutes*. Binding to chicken fibrinogen-domain containing proteins may result in the formation of random bacterial aggregates and those with the most optimal composition, e.g. butyrate producers releasing H_2_ with *Blautia* consuming H_2_ for acetate production thus allowing glycolysis in butyrate producers to proceed [16], will rapidly multiply and define final microbiota composition. Except for fibrinogen binding, representatives from the phylum *Bacteroidetes* had a greater potential to affect host behaviour than representatives from the phylum *Firmicutes*. *Bacteroidetes* encoded collagenase, hemagglutinin, hemolysin, hyaluronidase, heparinases, chondroitinase or mucin-desulfating sulfatase required for degradation of host structures. In addition, representatives from the phylum *Bacteroidetes* encoded endothelin-converting enzyme 1 precursor, which plays a significant role in cardiovascular diseases and Alzheimer’s disease in humans [41], 3-oxo-5-α-steroid 4-dehydrogenase capable of modification of steroid hormones or bile [42], or glutamate decarboxylase catalysing production of γ-aminobutyrate (GABA), a mediator within the enteric nervous system [43]. However, it will have to be elucidated whether these genes are expressed in vivo and whether their products may reach host structures.

Finally, the rather counterintuitive conclusion came from the prediction of survival following air exposure. Although *Bacteroidetes* should be more sensitive to air exposure than *Firmicutes* which are capable of spore formation, our data clearly showed that *Clostridiales*, including all important butyrate producers, were highly sensitive to air exposure. Due to the experimental design, we likely determined sensitivity of vegetative cells and not of spores. Despite this, the extreme sensitivity of vegetative cells of *Clostridiales* may explain their commonly reported disappearance in inflammatory bowel disease patients [44]. Inflammation leads to locally increased oxygen levels due to the oxidative burst of granulocytes and macrophages [45, 46]. Disappearance of *Clostridiales* including major butyrate producers can therefore be a consequence rather than a cause of inflammatory bowel diseases. Similarly, a reported increase in the abundance of *Bacteroidetes* or *Megasphaera* during inflammatory diseases [35, 47] may be a mere consequence of their higher resistance to oxygen and the disappearance of oxygen sensitive bacterial species from order *Clostridiales*. An overgrowth of facultative anaerobes like those from family *Enterobacteriaceae* in acute colitis also fits in the proposed scenario [47–49].

## Conclusions

In this study we isolated and sequenced 133 different strains originating from chickens intestinal tracts belonging to seven different phyla. Analysis of their genomic sequences showed that butyrate production was mainly associated with *Ruminococcaceae*, and less frequently with *Lachnospiraceae* or *Erysipelotrichaceae*, all belonging to phylum *Firmicutes*. Representatives of phylum *Bacteroidetes* commonly encoded proteins (collagenase, hemagglutinin, hemolysin, hyaluronidase, heparinases, chondroitinase, mucin-desulfating sulfatase or glutamate decarboxylase) that may enable them to interact with their host. Even such a brief list of genes shows that representatives of *Bacteroidetes* and *Firmicutes* follow different strategies of gut colonisation which contributes to their coexistence in the intestinal tract.

## Methods

### Ethics statement

The handling of animals in this study was performed in accordance with current Czech legislation (Animal Protection and Welfare Act No. 246/1992 Coll. of the Government of the Czech Republic). Experiments performed in this study were approved by the Ethics Committee of the Veterinary Research Institute (permit number 4/2016) followed by the Committee for Animal Welfare of the Ministry of Agriculture of the Czech Republic.

### Isolation and identification of caecal bacteria

The chickens were sacrificed under chloroform anesthesia by cervical dislocation. Whole caeca with their contents originating from 18 random healthy chickens or hens 4 to 40 weeks of age were removed during necropsy, chilled on ice and transported to an anaerobic chamber for further processing within one hour. The caeca were opened in an anaerobic chamber (10% CO_2_, 5% H_2_ and 85% N_2_ atmosphere; Concept 400, Baker Ruskinn, USA) and 0.5 g of content was squeezed into 4.5 ml pre-reduced PRAS dilution blank (0.1 g magnesium sulfate heptahydrate, 0.2 g monobasic potassium phosphate, 0.2 g potassium chloride, 1.15 g dibasic sodium phosphate, 3.0 g sodium chloride, 1.0 g sodium thioglycolate, 0.5 g L-cysteine, 1000 ml distilled water; final pH 7.5 +/− 0.2 at 25 °C) and mixed thoroughly. All samples were serially diluted in pre-reduced PRAS dilution blank and plated on Wilkins-Chalgren anaerobe agar (WCHA) (Oxoid) supplemented with 30% of rumen fluid. The rumen fluid was collected from cows by an oral probe, filtered through cheesecloth, centrifuged at 8000 g for 30 min and sterilised by filtration through a 0.22 μm filter. Aliquots of rumen fluid were stored at − 20 °C. WCHA was additionally supplemented with 5 mg/l hemin, 1 mg/l cellobiose, 0.5 g/l soluble starch, 1 mg/ml maltose, 0.2 ml vitamin K1 solution (0.1 ml of filter sterilized vitamin K1 in 20 ml 95% ethanol) and 0.5 mg/ml L-cysteine. Approx. 10 well-separated colonies of different morphology were selected from each agar plate after a five-day incubation at 37 °C and purified by subculture on WCHA. All isolates were stored at − 80 °C in pre-reduced PRAS dilution blank containing glycerol at 20% concentration and equal volume of sterile sheep blood. Sensitivity of pure anaerobe cultures to air oxygen exposure was tested exactly as described elsewhere [6]. Briefly, bacterial cultures were serially diluted in anaerobic chamber and plated on 4 copies of WCHA. One copy of WCHA was left in the anaerobic chamber to determine initial counts of each anaerobe. The remaining 3 copies of WCHA plates were placed into a standard aerobic 37 °C incubator and after 1, 8 and 24 h, a single copy of agar plate was returned back to the anaerobic chamber to check for growth restoration.

### Whole genome sequencing

DNA was purified using DNeasy Blood & Tissue Kit (Qiagen). Sequencing library was prepared from 1 ng of RNA-free genomic DNA using the Nextera XT DNA Sample Preparation kit (Illumina) and whole genome sequencing was performed using the NextSeq 500/550 High Output Kit v2 and Illumina NextSeq 500 sequencing platform in the paired-end modus (2 × 150 bp). Raw sequencing reads were quality trimmed using Trimmomatic v0.32 [50] with the sliding window of 4 bp and average quality threshold value equal to 17. Minimal read length was set to 48 bp. Trimmed paired-end reads were assembled via de novo assembler IDBA-UD v1.1.1 [51] with k-mer sizes ranging from 20 to 110 with an increment of 15. Contigs with coverage lower than 10% of average coverage of L50 contigs were filtered out and the remaining contigs were scaffolded employing SSPACE scaffolder v3.0 [52, 53]. All scaffolds containing N’s in their sequences were split into N-free sequences. Finally, scaffolds with a length shorter than 500 bp were discarded.

### Species definition and genome annotation

Species definitions used in this study are based on the BLAST comparison of whole 16S rRNA sequences against entries deposited in NCBI 16S rRNA sequence database performed on January 4, 2017. For clarity of the paper, we used the designation of the most similar bacterium based on the lowest E-value for description of our isolates, thus apparently ignoring the fact that in some cases there was 100% identity whilst in the opposite extreme, the sequence of 16S rRNA of one of our isolates was only 83% similar to the closest relative deposited in the NCBI 16S rRNA database (Additional file 1). All 16S rRNA sequences were compared also against RDP SeqMatch database (on January 10, 2017) which allowed for alternative taxonomy including classification of individual isolates into higher taxonomic units. In addition, ribosomal protein multilocus sequence typing (rMLST) [54] and GTDB organism identification (http://gtdb.ecogenomic.org/) databases were used to verify taxonomic classification. Taxonomic classification by these alternative protocols is provided in Additional file 1. Gene predictions and functional annotations were performed by RAST [55]. Assembled and annotated genomes as well as raw sequencing data were deposited in NCBI under accession number PRJNA377666 and genomes with comprehensive RAST annotation are available upon request.

### Genome comparison

To exclude genomes of the same isolates picked up on independent occasions from the subsequent analyses, whole genome sequences of all isolates were mutually compared using the QUAST tool v3.1 [56]. Two genomes were considered as identical if they shared more than 99% of genome content and had less than 1 indel per 100 kb. A single isolate was selected as a representative of each group of identical isolates for all downstream analyses.

Whole gene content similarity clustering was compared using Gene Co-Expression Network analysis. This protocol detects genes with similar transcriptional regulatory program (potential members of the same pathway, protein complex, etc.). In our case, the gene expression vector for particular gene was replaced by gene copy-number vector for particular bacteria and the protocol therefore detected bacterial isolates with similar gene content. Interconnected bacteria shared more than 50% of genes based on gene name designation provided by RAST annotation. Whole gene content similarity of individual isolates was analysed in R. At first, matrix of Spearman’s correlation coefficients was calculated for all pairs of isolates using vectors of respective gene counts. The correlation matrix was then transformed to the adjacency matrix using threshold of + 0.5 as a cut-off for two vertices to be considered as connected. The undirected network was constructed from such a matrix using igraph package (http://igraph.org/r/), and edge betweenness based community structure detection algorithm was then employed to identify separate network modules. Communities with more than three members were considered nontrivial and were highlighted in a network plot.

### Analysis of 16S rRNA genes

Trimmed reads were aligned against SILVA bacterial 16S rRNA database using SortMeRNA v2.1 [57] and extracted 16S rRNA reads were assembled via de Bruijn graph-based de novo assembler SPAdes v3.6.0 [58]. Finally, sequences coding for 16S rRNA genes were predicted employing barrnap tool v0.6 (https://github.com/tseemann/barrnap). For the purposes of phylogenetic analysis, 16S rRNA genes were aligned by ClustalW v2.1 [59] using default gap penalties, DNA weight matrix IUB and transition weight 0.2. The phylogenetic tree topologies were inferred employing Bayesian statistics via MrBayes v3.2.6 [60] using the parameters as follows: mixed model of nucleotide substitution, gamma-distributed rates among sites, four Monte Carlo Markov chains for 7,000,000 cycles which were sampled every 1000th generation and the first 25% of the samples were discarded as burn-in. The final tree topology was generated employing 50% majority-rule consensus. Average standard deviation of split frequencies was 0.0083, maximum standard deviation of split frequencies was 0.1095, average potential scale reduction factor was 1.000 and maximum potential scale reduction factor was 1.015. The final tree topology was visualized by iTOL v3.4.3 [61] and edited using Inkscape v0.91 (www.inkscape.org).

## Additional files

Additional file 1: List of 133 different isolates characterised in this study. The file contains taxonomical classification using NCBI and RDP databases based on whole sequence of 16S rRNA, similarity to the closest relative in the NCBI database, genome size, number of contigs into which the genome was assembled, genomic and 16S rRNA GC content, consensus 16S rRNA sequences with SNP positions indicated with lower case letters, 16S rRNA copy number estimated based on sequencing coverage and 16S rRNA copy number determined by RT PCR (only for the isolates in which sequencing coverage predicted more 10 copies of 16S rRNA genes). (XLSX 338 kb)

Additional file 2: Phylogenetic tree of 133 sequenced isolates obtained from chicken caecum based on the Clustal alignment of the full-length sequence of RpoB proteins. Families within the phylum *Firmicutes* are shown in light blue, green and yellow. Families within the phylum *Bacteroidetes* are shown in shades of purple. The whole genome size and genomic GC content of each isolate is shown external to the dendrogram. (PDF 576 kb)

Additional file 3: Aerobic survival of chicken gut anaerobes. (XLS 43 kb)

Additional file 4: List of proteins encoded by individual anaerobes. (XLSX 7930 kb)

Additional file 5: Distribution of genes in selected categories among representatives of major gut colonisers belonging to phyla *Bacteroidetes* and *Firmicutes.* X axes indicate the numbers of genes in a given category per genome. (PDF 23 kb)

## Abbreviations

ABC transporter: ATP-binding cassette transporter
*batABCDE* operon: *Bacteroides* aerotolerance operon
BUK: Butyrate kinase
ECF class transporter: Energy-coupling factor transporter
GABA: γ-aminobutyrate
GTDB: Genome taxonomy database
NCBI: National Center for Biotechnology Information
OTU: Operational taxonomic unit
PBT: Phosphate butyryltransferase
PRAS dilution blank: Pre-reduced anaerobically sterilized dilution blank
RAST: Rapid annotations using subsystems technology
RDP: Ribosomal database project
rMLST: Ribosomal protein multilocus sequence typing
TRAP transporter: Tripartite ATP-independent periplasmic transporter
WCHA: Wilkins-Chalgren anaerobe agar
