# Supplementary material for: Whole genome sequencing and function prediction of 133 gut anaerobes isolated from chicken caecum in pure cultures
Source: BMC Genomics. 2018 Jul 31;19:561. doi: 10.1186/s12864-018-4959-4 (PMC6069880; doi:10.1186/s12864-018-4959-4)
Supplement: Supplementary file 2 — Phylogenetic tree of 133 sequenced isolates obtained from chicken caecum based on the Clustal alignment of the full-length sequence of RpoB proteins. Families within the phylum Firmicutes are shown in light blue, green and yellow. Families within the phylum Bacteroidetes are shown in shades of purple. The whole genome size and genomic GC content of each isolate is shown external to the dendrogram. (PDF 576 kb) [file 12864_2018_4959_MOESM2_ESM.pdf]

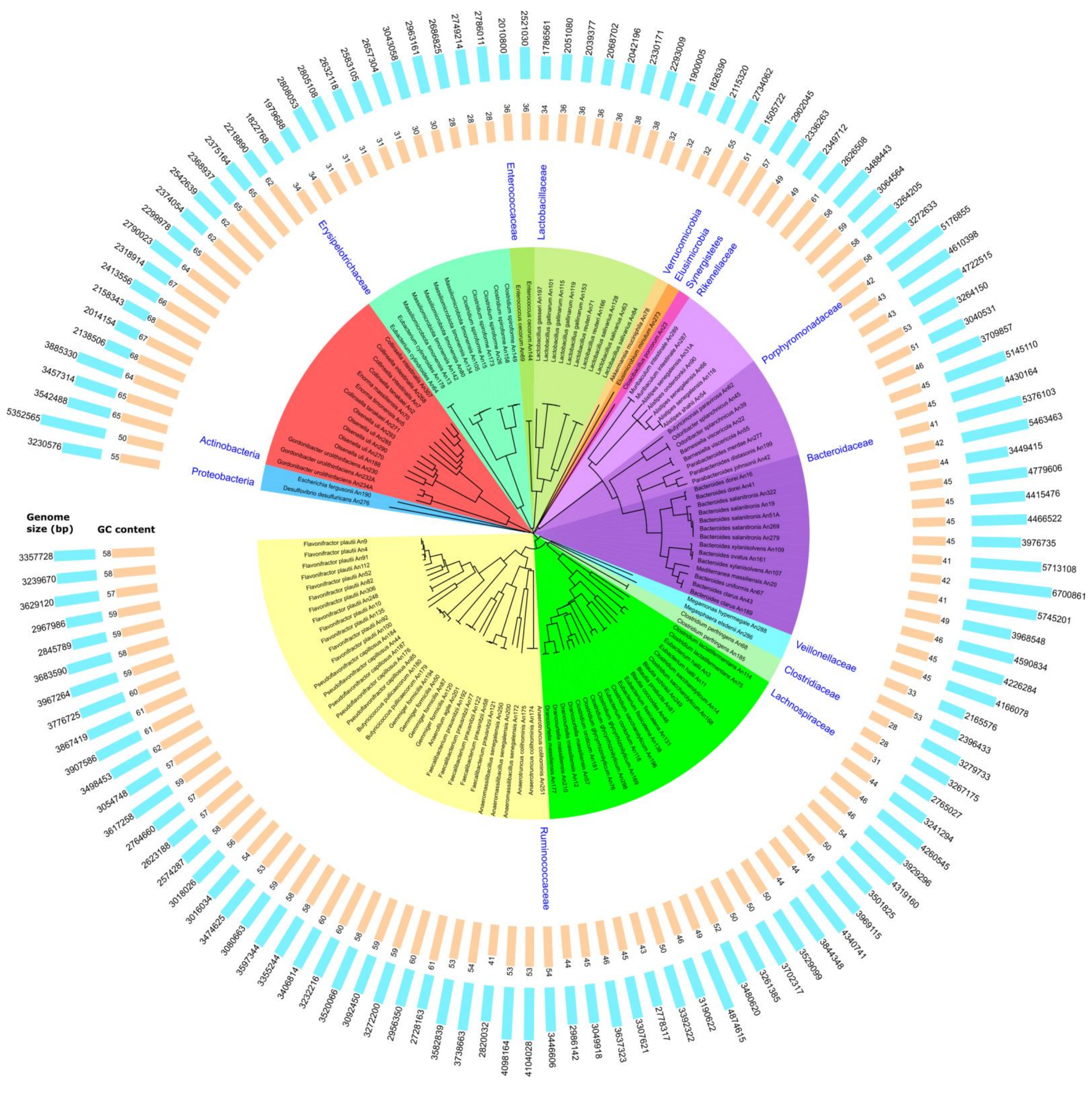

Additional file 2. Phylogenetic tree of 133 sequenced isolates obtained from chicken caecum based on the Clustal alignment of the full-length sequence of RpoB proteins. Families within the phylum *Firmicutes* are shown in light blue, green and yellow. Families within the phylum *Bacteroidetes* are shown in shades of purple. The whole genome size and genomic GC content of each isolate is shown external to the dendrogram.
