# Supplementary figures and images for: Whole genome sequencing and function prediction of 133 gut anaerobes isolated from chicken caecum in pure cultures
Source: BMC Genomics. 2018 Jul 31;19:561. doi: 10.1186/s12864-018-4959-4 (PMC6069880; doi:10.1186/s12864-018-4959-4)

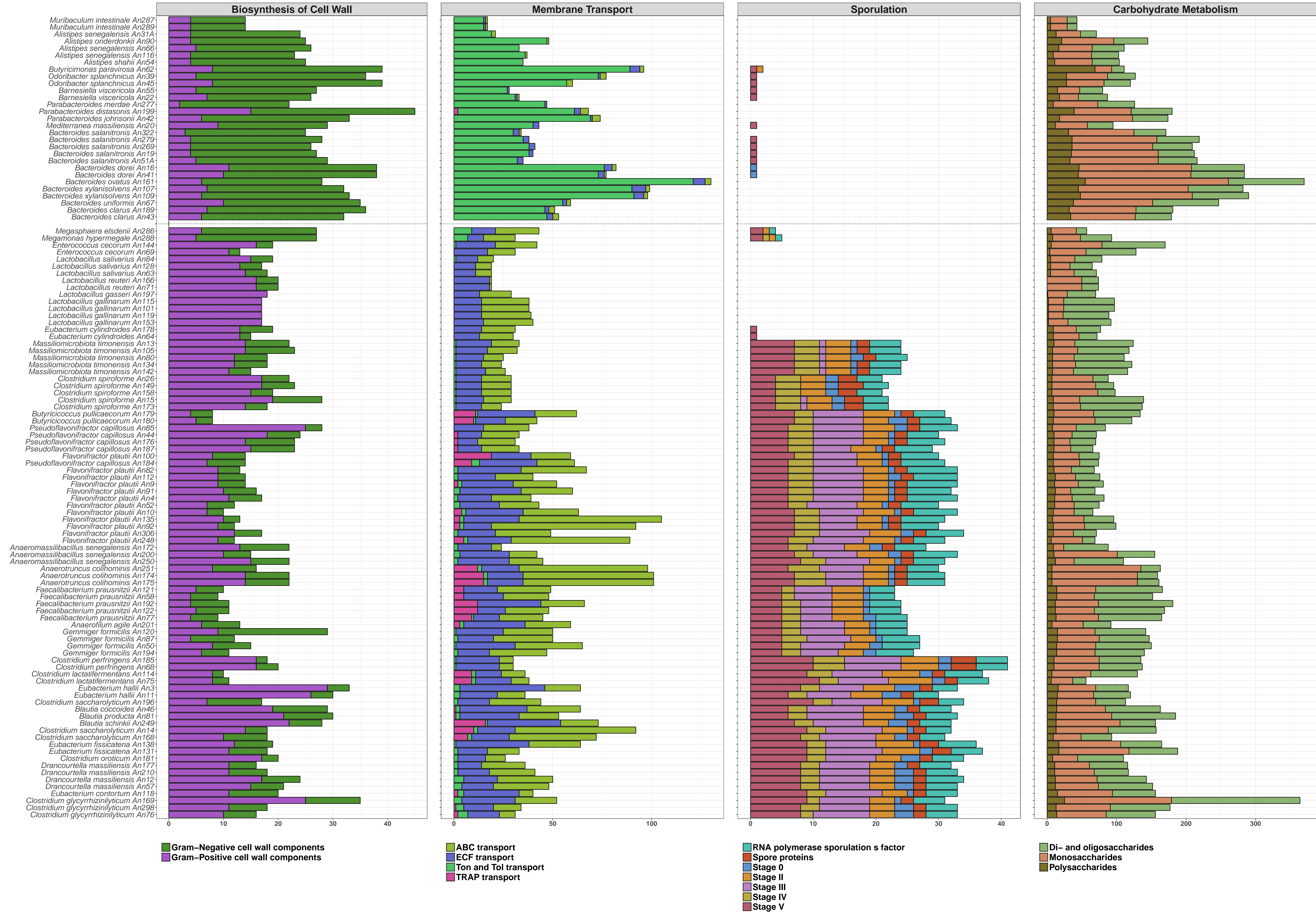

Supplement: Supplementary file 5 — Distribution of genes in selected categories among representatives of major gut colonisers belonging to phyla Bacteroidetes and Firmicutes. X axes indicate the numbers of genes in a given category per genome. (PDF 23 kb) [file 12864_2018_4959_MOESM5_ESM.pdf]
